# Quantitative assessment of depolarization by the retinal pigment epithelium in healthy and glaucoma subjects measured over a large field of view

**DOI:** 10.1371/journal.pone.0278679

**Published:** 2022-12-13

**Authors:** Alice R. Motschi, Florian Schwarzhans, Sylvia Desissaire, Stefan Steiner, Hrvoje Bogunović, Philipp K. Roberts, Clemens Vass, Christoph K. Hitzenberger, Michael Pircher

**Affiliations:** 1 Center for Medical Physics and Biomedical Engineering, Medical University of Vienna, Vienna, Austria; 2 Center for Medical Statistics, Informatics and Intelligent Systems, Medical University of Vienna, Vienna, Austria; 3 Department of Clinical Pharmacology, Medical University of Vienna, Vienna, Austria; 4 Department of Ophthalmology and Optometry, Medical University of Vienna, Vienna, Austria; 5 Christian Doppler Laboratory for Artificial Intelligence in Retina, Medical University of Vienna, Vienna, Austria; Nicolaus Copernicus University, POLAND

## Abstract

We present measurements of depolarization introduced by the retinal pigment epithelium (RPE) over a 45° field of view using polarization sensitive optical coherence tomography. A detailed spatial distribution analysis of depolarization caused by the RPE is presented in a total of 153 subjects including both healthy and diseased eyes. Age and sex related differences in the depolarizing character of the RPE are investigated.

## Introduction

The retinal pigment epithelium (RPE) is a pigmented mono-cellular layer located anterior to the choroid and posterior to the neural retina. Together with the neural retina, the RPE is part of the retina, and the melanin therein contained protects ocular cells by absorbing light and acting as an anti-oxidant [1]. In addition, the RPE supports the metabolism of the entire retina and is responsible for the phagocytosis of the photoreceptor outer segment membrane disks that are potentially harmed by photo-oxidative stress [2,3]. It could be shown that RPE dysfunction leads to photoreceptor dysfunction and finally to a loss of vision [1]. This dysfunction has been linked to retinal diseases such as age-related macular degeneration [4].

The RPE can be visualized non-invasively and in vivo by using optical coherence tomography (OCT), where it appears in cross sectional images of the retina as strongly backscattering layer [5,6]. These images can be used for a detection of anomalies in the RPE. However, conventional OCT doesn’t always allow for clear distinction between the boundaries of the RPE and other strongly reflecting layers nearby, such as the interdigitation zone of the interface between cone photoreceptors and the RPE (or end tips of photoreceptors), and the boundary between inner and outer photoreceptor segments. More specifically, if the RPE is disrupted due to a retinal disease, the position and appearance might change, making it difficult to separate it from other structures such as for example sub-retinal hyper-reflective material (SHRM) [7]. However, pigments such as melanin that are contained in the RPE cells have the property to depolarize backscattered light while other outer retinal layers such as the photoreceptor layers are mainly polarization preserving. A differentiation between these retinal tissue properties can be performed by polarization-sensitive (PS) OCT [8,9], which represents a functional extension of the standard OCT technique and allows for a clear and selective visualization of RPE tissue [10–16]. In vitro studies have shown that the amount of depolarization caused by melanin depends on its concentration [17,18].

Depolarizing tissue can be characterized by quantifying local fluctuations of the measured polarization state returning from the depolarizing tissue. This can be done by calculating a measure that is related to the classical degree of polarization: the degree of polarization uniformity (DOPU) [19]. DOPU is a unit-less value between 0 and 1, where strongly depolarizing tissues have low DOPU values while polarization preserving tissues have DOPU values close to 1. As DOPU depends on the input polarization state that is not always known in various PS-OCT instrumentations, other metrics such as depolarization index [20] or entropy [18] have been introduced as well to describe the depolarizing character of tissue.

Previous studies have shown in a limited field of view that the depolarizing capability of the RPE in healthy eyes varies with retinal location such that depolarization is strongest in the macula area (excluding the foveola) and decreases towards the periphery [21,22]. At the foveola, depolarization appeared to be less strong than in the surrounding macular area [22]. The varying depolarization strength might be explained by the RPE’s anatomy: RPE cells in the macular area are taller and narrower, and therefore melanin pigments are packed more densely in this region, causing a higher local melanin concentration [23]. Furthermore, it was recently shown that in an Asian population, the RPE becomes more depolarizing with increasing age [21], possibly due to a change of pigmentation concentration with age [24,25].

In the present work, we expand previous spatial depolarization studies of the RPE to a larger field of view, equivalent to the size of a standard (45°) fundus photography and a larger (Caucasian) study population, spanning several decades of age. For the first time, we include diseased (glaucomatous) eyes into the analysis, where glaucoma is a group of neurodegenerative diseases progressively leading to retinal ganglion cell death, causing vision loss [26]. Finally, we present a detailed spatial distribution of depolarization caused by the RPE and provide an investigation of possible age and sex related changes in the depolarizing character of the RPE.

## Methods

### PS-OCT imaging

PS-OCT data was recorded with a custom-built spectral domain PS-OCT system based on a Michelson interferometer using polarization maintaining (PM) fibers and a single, circular polarized input state that is incident onto the eye [27]. The system comprises an integrated retinal tracker using line scanning laser ophthalmoscopy (LSLO) to correct for involuntary eye movements. The OCT sampling beam is generated by a superluminescent diode with a center wavelength of 860 nm, a bandwidth of 60 nm, and an A-scan rate of 70 kHz. At the same time, the retina is scanned at 60 Hz with a line-shaped LSLO beam with a wavelength of 786 nm and a beam power of 0.7 mW [27]. To fulfill laser safety regulations for the combined beams [28], the OCT beam is kept at a power of 0.5 mW. The system attains an axial resolution of 4.2 μm in tissue, a lateral resolution of ~20 μm, and a sensitivity of 98 dB [27].

**Fig 1:**
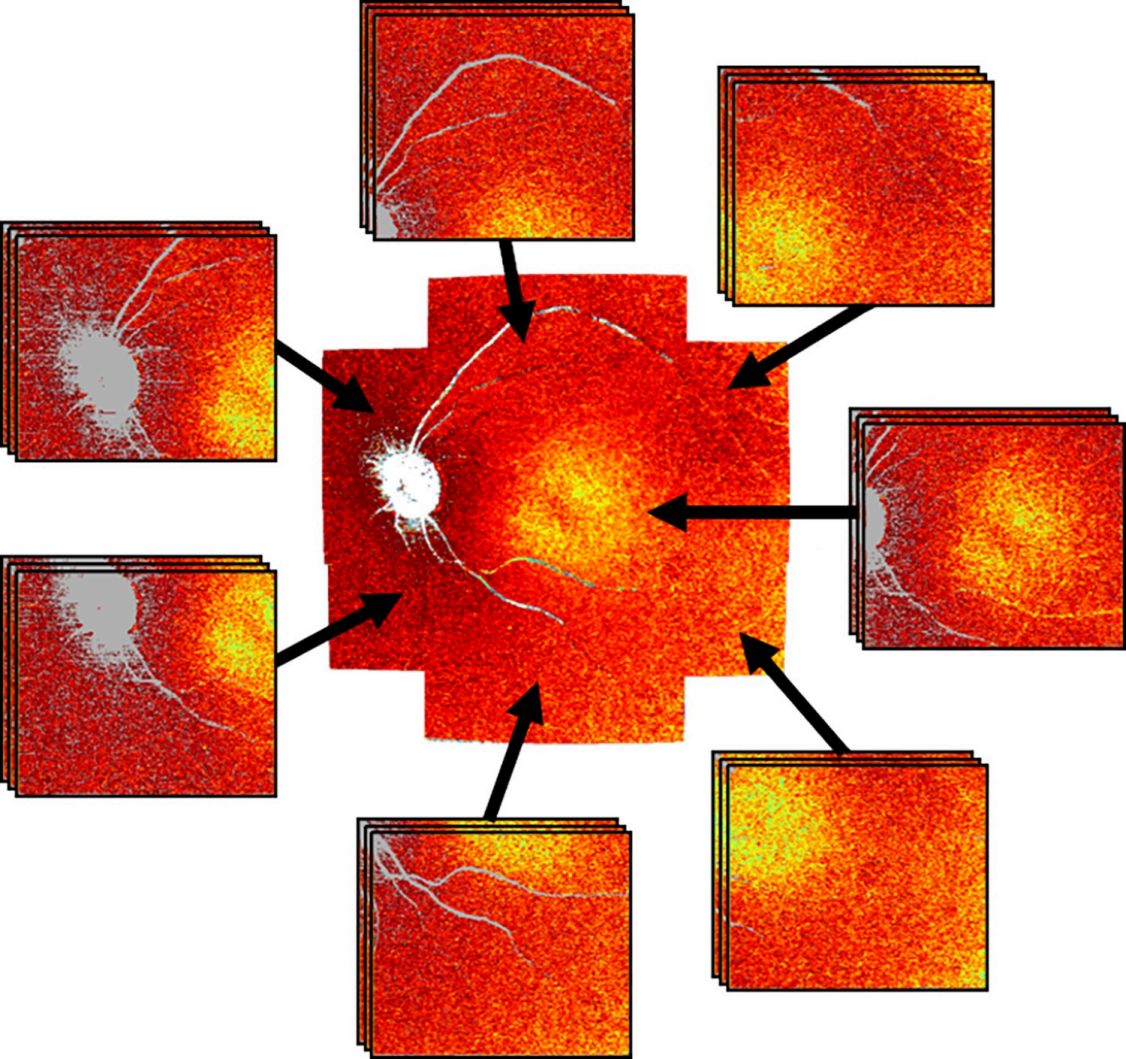
Illustration of the used scanning pattern showing seven distinct imaging positions (sub-fields). Each subject was measured three times each at seven distinct positions. The resulting 21 datasets were combined to a large field, noise reduced dataset.

Each subject was imaged three times (on two different study days), each at seven distinct positions (sub-fields): one centered at the macula and six evenly distributed around the macula, resulting in 21 volume datasets in total per subject (cf. Fig 1) [29]. Each of the 21 datasets consists of 250 B-scans comprising 1024 A-scans and covers a region of size 28° (x) by 21° (y), corresponding to an area of 8 × 6 mm^2^ on the retina in a standard eye. Acquisition time of a single dataset was 4.5 s. The 21 datasets recorded in each subject were then combined to a large field, noise reduced dataset, as described below.

### Subjects

105 eyes of 105 healthy volunteers of age 50.1 ± 16.6 years (mean ± SD, range: 21.1 to 78.9 years) were included into the study. 58 of the healthy volunteers were female, 47 were male. In addition, 48 patients with glaucoma at an early stage with the following subtypes were included: primary open angle glaucoma (40 patients), pseudoexfolliative glaucoma (3 patients), juvenile glaucoma (2 patients), pigmentary glaucoma (2 patients), and chronic angle closure glaucoma (1 patient). Patients and healthy volunteers were screened and included in this study by glaucoma specialists of the Medical University of Vienna. Early glaucoma was defined in our patients as glaucomatous appearance of the optic nerve head and repeated pathologic 24–2 visual field tests with a mean deviation of −6 dB or better. Patients with evidence of other ocular disease were not included in our analysis. The included patients’ age ranged from 39.9 to 78.4 years (on average: 63.1 ± 9.9 years), 28 of them were female and 20 were male. This study was approved by the local ethics committee (approval number: EK-Nr. 1673/2017), adhered to the tenets of the Declaration of Helsinki and written informed consent was obtained from all participants.

### Data analysis

#### Standard processing of spectral domain 3D PS-OCT data

The two channels of the PS-OCT instrument recorded OCT data for each of the 250 B-scans per dataset. This data was processed using a standard protocol of spectral domain volume data processing to yield amplitude and phase of the corresponding OCT signals, from which PS-OCT data (retardation, DOPU, Stokes vectors) are derived. The processing includes the compensation of PM fiber length mismatch, as described earlier [30]. In each B-scan, pixels with intensity values below two times the intensity noise level were excluded and then, Stokes vectors were calculated from the OCT amplitude and phase data. DOPU values were computed as described in previous work [19] using a kernel size of 10 × 10 pixels (corresponding to approximately 20 μm [axial] × 78 μm [lateral]). For intra-retinal layer segmentation, a graph-based segmentation method (The Iowa Reference Algorithms, Retinal Image Analysis Lab, Iowa Institute for Biomedical Imaging, Iowa City, IA) [31,32], was used.

#### Calculation of large field of view depolarization maps

From the layer segmentation mentioned above we retrieved a segmentation for the RPE. Since this algorithm only considers the intensity data, the output segmentation lines might jump to other hyper-reflective areas and therefore yield erroneous segmentation results. Thus, the segmentation line of the RPE is corrected as follows: since the RPE is depolarizing, we assume that for each A-scan, the pixel within the DOPU image at the location of the RPE segmentation line has a low (< 0.6) DOPU value. Therefore, we determine batches of one or more consecutive pixels with low DOPU value in that A-scan, and select the batch that is closest to the segmentation line found by the algorithm. Because the DOPU value of a certain pixel is basically an average of its surroundings, the boundaries of the RPE might show a slightly higher DOPU value than the center of the RPE. Thus, we set the corrected segmentation line point within an A-scan to the position of the minimum DOPU value within the selected batch, assuming that this corresponds to the center of the RPE.

Having determined the location of the RPE, the DOPU value at the RPE segmentation line is extracted for the generation of depolarization en-face maps. In total, three sets (measurements) of en-face maps (sub-fields) from seven different retinal locations are obtained per subject. To reduce noise, the three measurements (maps) for each of the seven sub-fields are averaged. From the resulting averaged maps, a large field of view en-face map (covering ~45°, comparable to a standard fundus photography) was generated using a stitching algorithm that registers the maps against a reference fundus image and fuses them seamlessly [29]. Due to artifacts caused by the ONH, values in a radius of 140 pixels (~1.1 mm) around the center of the ONH were excluded.

#### Calculation of spatial depolarization distribution

**Fig 2:**
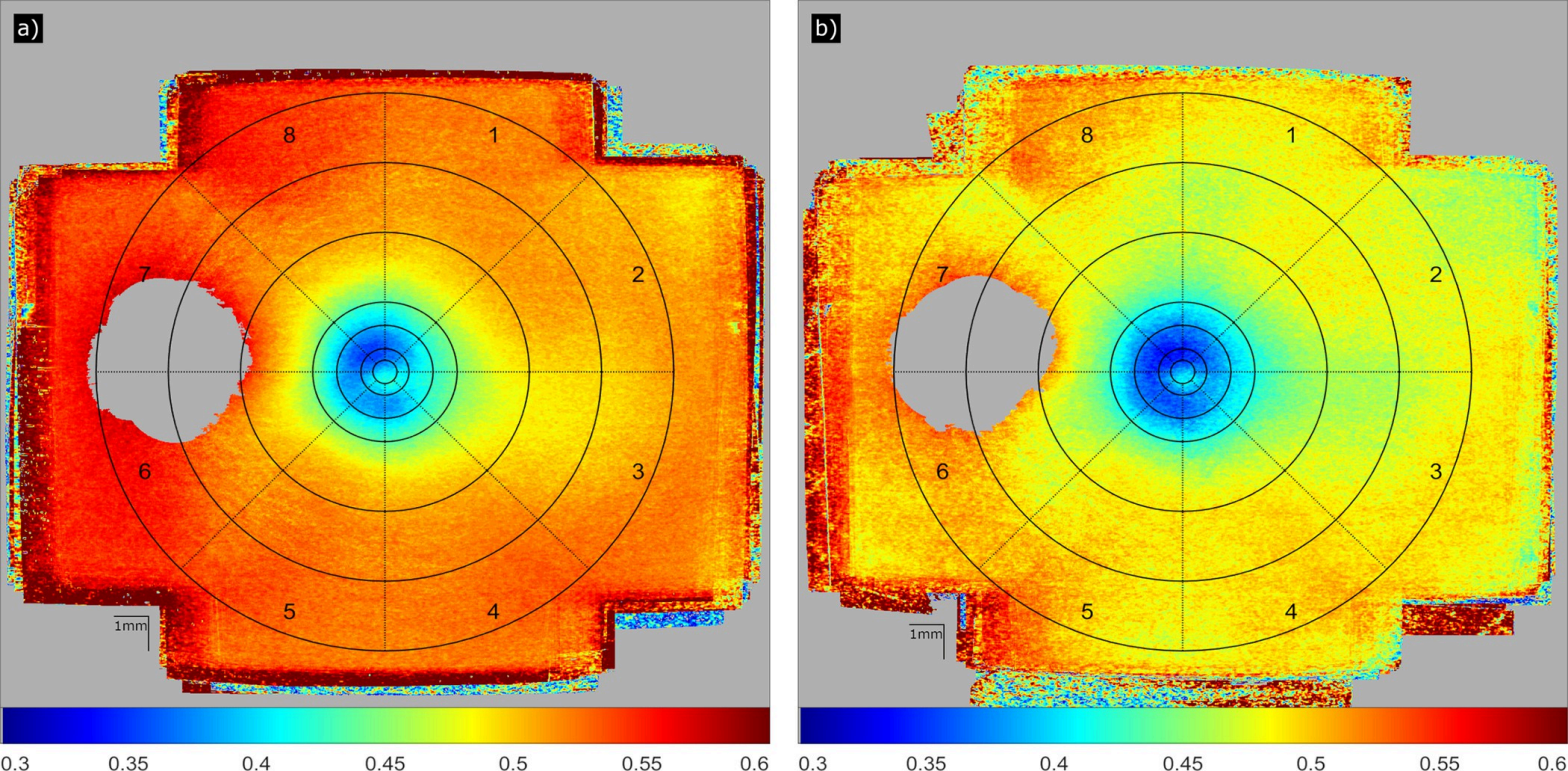
Depolarization caused by the RPE visualized by DOPU, averaged over 105 healthy volunteers (a) and 48 glaucoma patients (b), respectively. The grids are centered at the fovea and the diameter of the innermost circle (A) is 0.5 mm. The outer diameters of the surrounding rings (center to periphery: B, C, D, E, F, and G) are 1, 2, 3, 6, 9, and 12 mm, respectively. The sectors are numbered from 1 to 8. The color scheme corresponds to DOPU (low DOPU value [blue color] indicates strong depolarization), with gray indicating locations that have been excluded from the analysis.

To provide a quantitative comparison between healthy and glaucomatous subjects, ages and sexes across various retinal locations, a grid centered at the fovea was placed on the large field of view maps (cf. Fig 2). The grid was inspired by the chart proposed by the Early Treatment of Diabetic Retinopathy Study (ETDRS) [33] and was extended to accommodate both the larger field of view and the substantial variation of DOPU close to the fovea. It consists of an inner circle with a diameter of 0.5 mm, surrounded by six rings with diameters of 1, 2, 3, 6, 9, and 12 mm. The rings are equally divided into 8 sectors, resulting in 49 field locations in the grid. For each field location of the grid, mean DOPU values and standard errors were calculated.

## Results

Average DOPU en-face maps of all healthy volunteers and glaucoma patients, centered at the fovea, are shown in Fig 2(A) and 2(B), respectively. In both cases, the depolarization is strongest (DOPU is smallest) in the two rings around the center of the fovea (rings B and C), with a slightly weaker depolarization at the center of the fovea (ring A).

**Fig 3:**
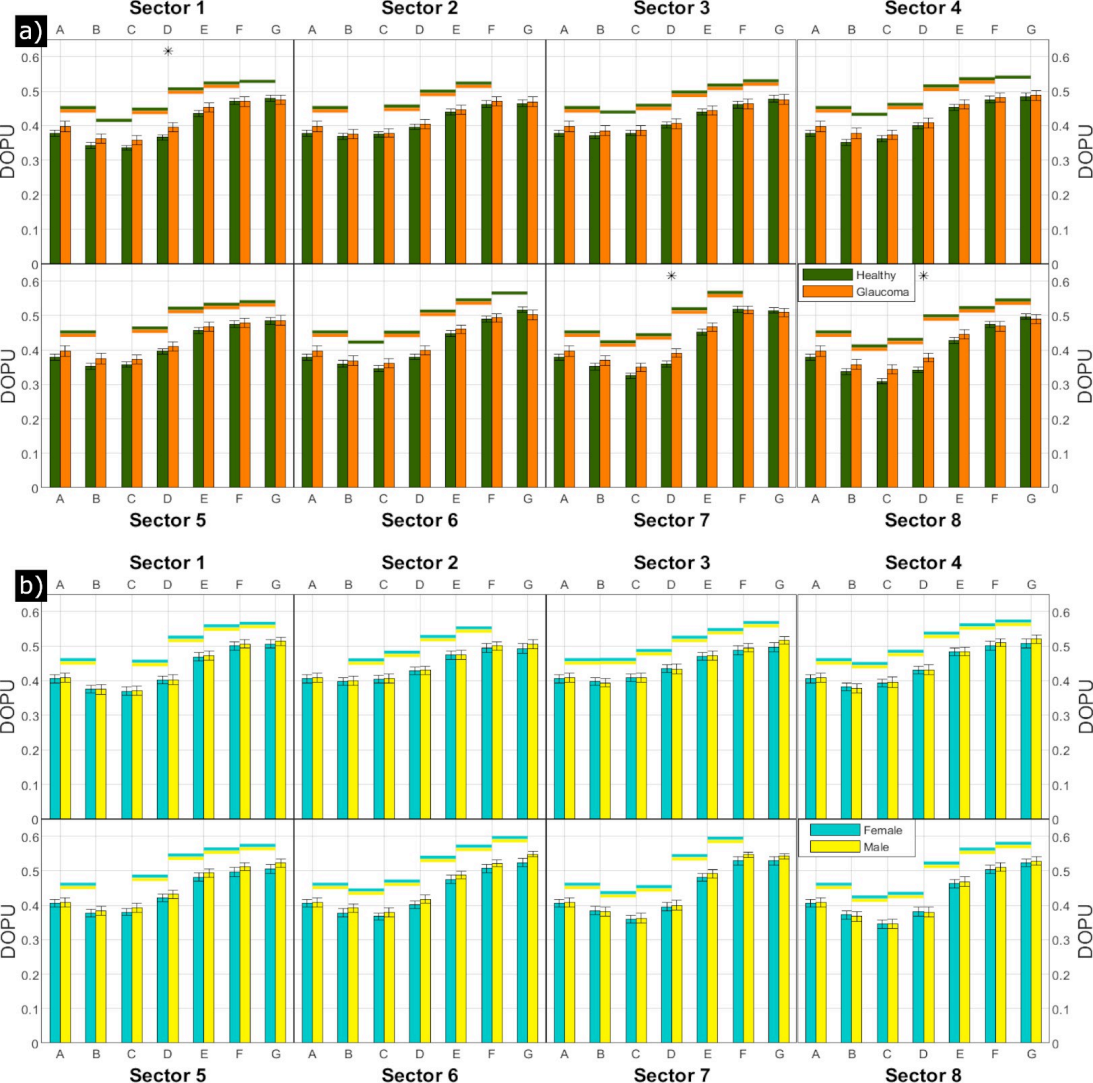
Quantitative analysis of DOPU values for different retinal locations. Each column represents averaged DOPU values for every ring per sector of the grid defined in Fig 2. A denotes the circle at the center, G the outermost ring. The error bars correspond to the standard error across subjects. The color-coded horizontal lines above two neighboring bars indicate that the differences between these fields are significant at 5%. (a) Average over 76 healthy volunteers (green) and over 48 glaucoma patients (orange). All subjects were 39 years old or older. An asterisk above a group of bars indicates that the difference between healthy volunteers and glaucoma patients in this field is significant at 5%. (b) Average over 58 female healthy volunteers (turquoise) and 47 male healthy volunteers (yellow). In no field location the difference between female and male was significant at 5%.

Towards the periphery, the depolarization strength decreases. This pattern is similar in healthy subjects and glaucoma patients, but in general, depolarization seems to be slightly more pronounced in glaucoma patients. However, this might be explained by the age difference between the two groups. Therefore, healthy subjects younger than 39 years were excluded for the following analysis (Fig 3(A)), since there were no age-matched glaucoma patients in our study group. In Fig 3(A), the mean values per grid location across the 76 healthy volunteers aged 39 years or older (green) and across all glaucoma patients (orange) were calculated and displayed in bar charts per sector. The leftmost bar (ring A) corresponds to the central circle and the error bars to the standard error. The green or orange line above two neighboring bars signifies that the difference between those two bars is statistically significant (Student’s t-test at 5% significance level) for the healthy volunteers or the glaucoma patients, respectively. Minimum DOPU (strongest depolarization), ranging between 0.31 and 0.39, was reached in the innermost rings next to the central circle (rings B, C), the weakest depolarization (DOPU ≈ 0.52) can be observed in the outermost ring (G). With a few exceptions, neighboring rings appear to be significantly different from each other. An asterisk above a bar group indicates that healthy volunteers and glaucoma patients are significantly different at 5% in this field. With a few exceptions in ring D, the difference between healthy volunteers and glaucoma patients is not significant.

Fig 3(B) shows the average DOPU of healthy volunteers, separated by sex (turquoise: female, yellow: male). Again, the colored lines above neighboring bars indicate statistical significance at 5% for these fields, which is the case for most of the pairs. Comparing sexes, the differences between female and male weren’t significant for any field location.

**Fig 4:**
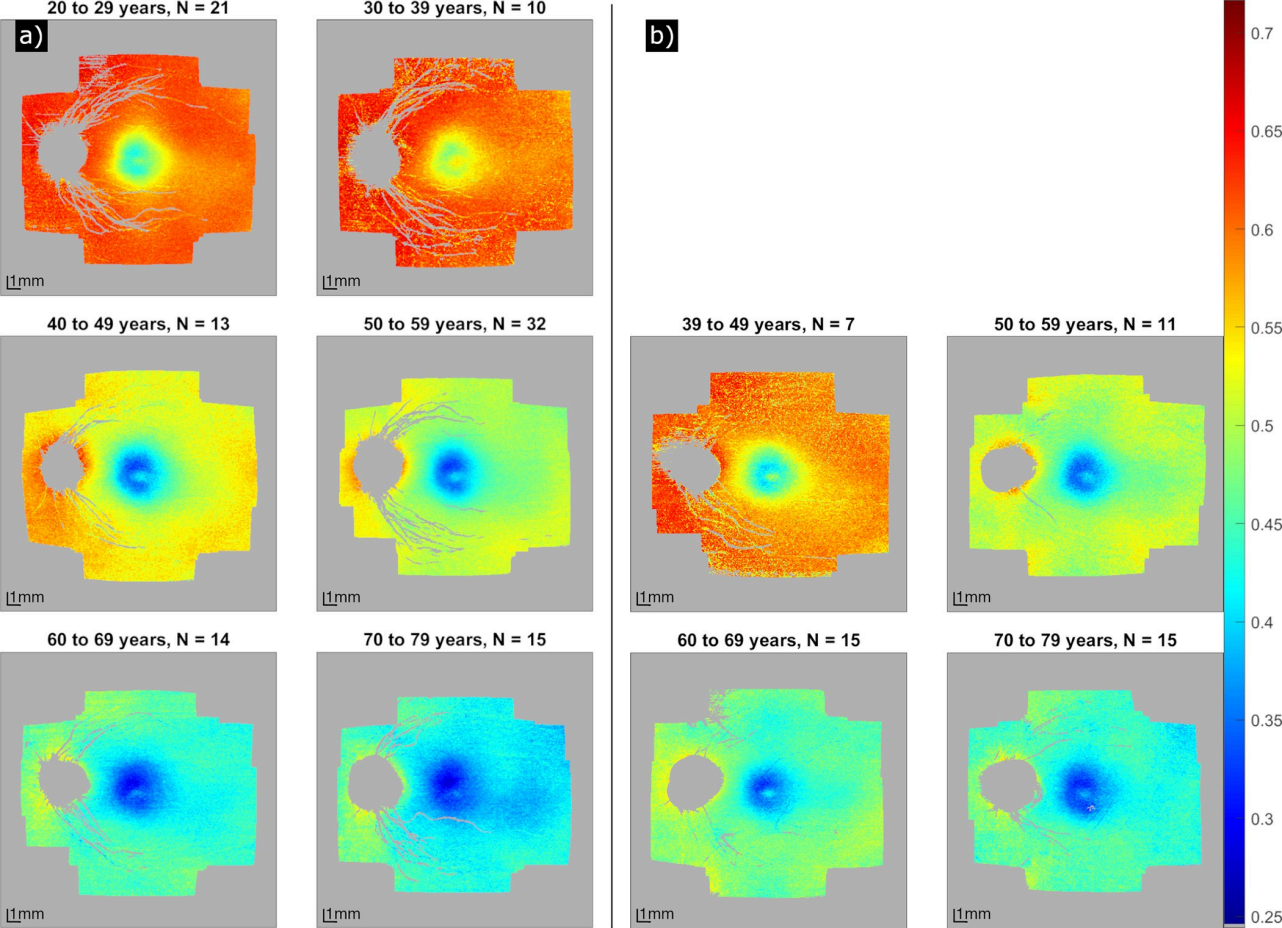
DOPU en-face maps in a large field of view of healthy volunteers (a) and glaucoma patients (b), for different age groups. The number N of participants per map is depicted in the heading. The color values correspond to DOPU (low values: Strong depolarization), gray values indicate pixels that have been omitted from the calculation.

**Fig 5:**
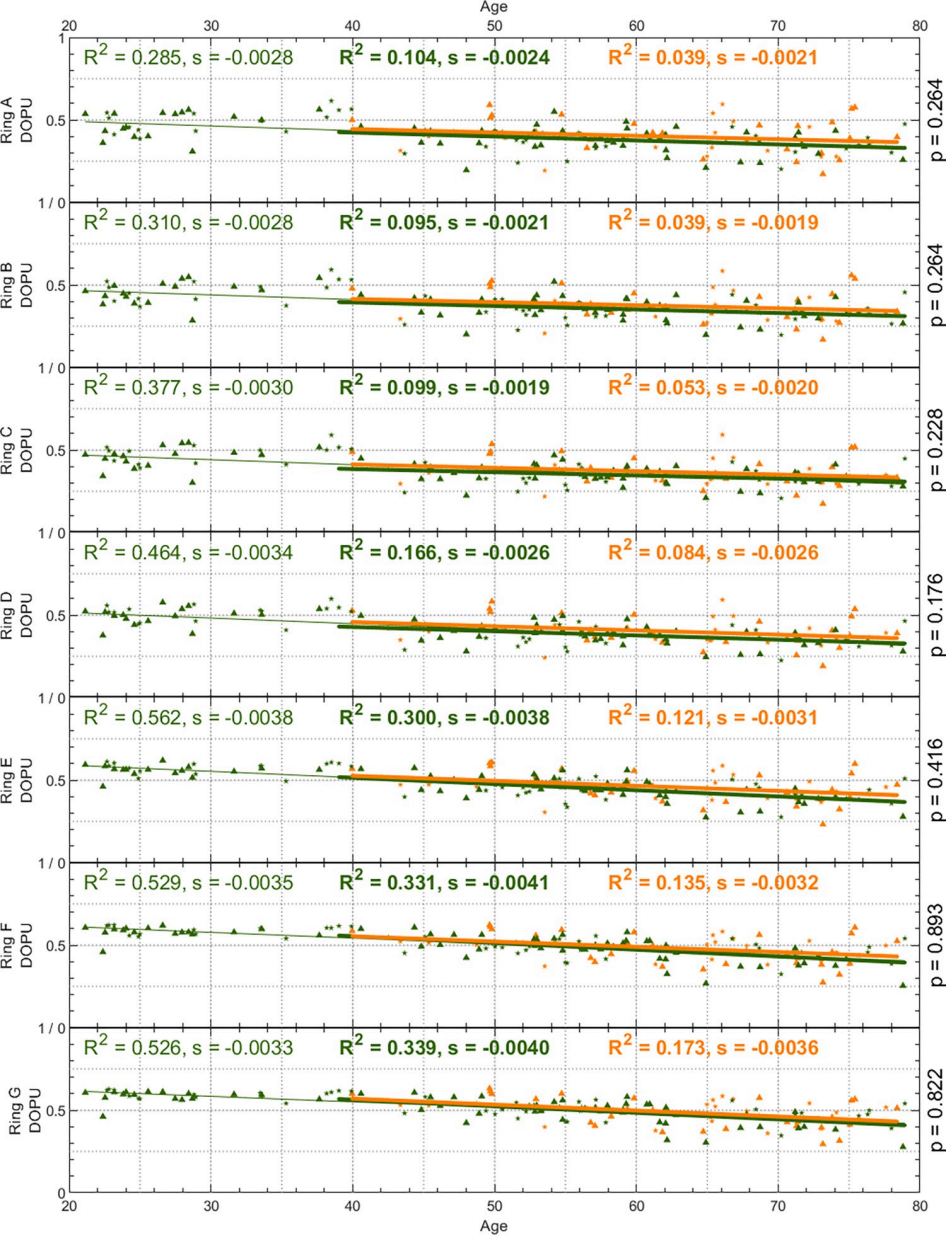
DOPU plotted against the age of the participants for each ring of the evaluation grid in Fig 2. Healthy volunteers are plotted in green (N = 105), glaucoma patients in orange (N = 48). Female participants are represented with a triangle, male participants with a star. For the healthy volunteers aged 39 years or older (bold) and glaucoma patients (bold) as well as for the healthy volunteers of all ages (normal), linear regression lines were plotted and the coefficients of determination (R^2^) and slopes (s) were calculated. On the right-hand side of each plot, the p-value of Student’s unpaired t-test between healthy volunteers and glaucoma patients is displayed.

Fig 4(A) and 4(B) show depolarization maps averaged per decade of age for healthy volunteers and glaucoma patients, respectively. It can be observed that DOPU decreases across the entire field of view with age (depolarization strength increases). A more detailed analysis is presented in Fig 5: for each ring (and the center circle), an average DOPU was calculated for each subject and plotted against the age of the subject. Healthy volunteers are displayed in green, glaucoma patients in orange. Female participants are represented with a triangle, male participants with a star. For healthy and glaucomatous subjects as well as for the subset of healthy volunteers older than 39 years individually, linear regression lines and the corresponding coefficients of determination (R^2^) and slopes (s) were calculated. The plot shows that with increasing age, DOPU decreases (depolarization strength increases), but there is a high variability for both healthy volunteers and glaucoma patients. The decrease in DOPU with age is similar for all rings and there is no significant difference between healthy and glaucoma subjects.

## Discussion

It has been shown that the RPE is depolarizing [10] and that its depolarization capability varies across the retinal location in the macula [21,22] and is distorted in myopia [34]. Thereby, the amount of depolarization depends on the age of the subjects as has recently been shown for an Asian study group [21]. We extended these previous findings in several ways.

First, a larger field of view was investigated, stitched together from seven slightly overlapping locations around the macula to achieve a total field of view of ~45° that as such matches the size of a standard fundus photography. In the macula, our study confirms the varying spatial distribution of depolarization [21,22]. In the peri-macular region we observed a continuation of the trend that depolarization decreases (DOPU increases) with eccentricity from the fovea. In addition, we could show that depolarization varies between different sectors of our evaluation grid (cf. Figs 2 and 3) that in total investigates the depolarization in 49 regions of interest (8 sectors in 6 rings plus the central circle).

Second, we substantially increased the number of Caucasian subjects (105 healthy volunteers) compared to our previous study [22]. In our study population we found a similar trend of increasing depolarization with age (cf. Fig 5) as has previously been shown in the Asian population [21]. The age dependency is similar for all eccentricities from the fovea. Thereby, it must be noted that the previous study used the entropy as quantitative measure for depolarization while our approach relied on DOPU. The similarity of the results indicates that depolarization may similarly be described by both quantities. It has been shown, however, that DOPU depends on the incident polarization state (with lowest DOPU values for circular polarized light and largest DOPU values for linear polarized light) [20] and although our system emits a circular polarization state, birefringent structures such as the cornea, the retinal nerve fiber layer or Henle’s fiber layer may alter this polarization state.

However, birefringence introduced by the cornea is quite low and generated a retardation in the range between 10° and 36° which corresponds to an elliptical polarization state that is closer to a circular state than to a linear state and as such the influence on the corresponding DOPU measurements will be low. In order to test this hypothesis, we compared age-matched DOPU measurements in subjects showing low corneal birefringence (retrieved from retardation values measured at the surface of the retina) with subjects showing high corneal birefringence. In the first group we determined a range of retardation values measured at the retinal surface between 10° und 17° while in the second group we determined a range between 25° und 36°. We found no significant difference in the DOPU measurements between these two groups in all sectors apart from sector 1, rings F and G, which supports our hypothesis of little influence of corneal birefringence on the presented DOPU measurements. Nevertheless, more care is needed for DOPU calculation in instruments that do not use a well-defined circular polarization input state or in imaging scenarios where the light passes through highly birefringent media.

Third, we included for the first time pathological eyes, in our case 48 early-stage glaucoma patients. In this early stage of the disease, no changes in depolarization could be found in comparison to healthy eyes (cf. Fig 3(A)) and both groups show a similar age dependency (cf. Fig 5). The small difference in the appearance of the averaged DOPU images (cf. Fig 2) may be explained by the difference in the average age of the two groups. The preservation of RPE depolarization might be explained by the nature of glaucoma as a neuronal disorder that first affects the retinal nerve fiber layer and only in a later stage outer retinal structures such as photoreceptors and the RPE might be affected to some degree [35]. Given that the patients included into this study were still at an early stage of the disease, we cannot exclude that potential deteriorations are present in a later stage. However, such investigation requires to repeat the study with patients at a more advanced stage of glaucoma. Generally speaking, the study is merely a start and can be repeated with other diseases known to affect the RPE, such as age-related macular degeneration at an early stage.

Finally, we investigated potential sex-related differences of depolarization in the RPE. Here we did not find any statistically significant difference between the two groups (cf. Fig 3(B)).

The observed spatial distribution of DOPU is most likely caused by the presence of pigments within the RPE such as melanin, melanolipofuscin, and melanolysosomes [36]. As suggested previously [21], the increase of polarization strength with age can most likely be associated with an age-induced change of pigment composition. The concentration of lipofuscin, as well as complex types of melanin (melanolipofuscin and melanolysosome), increase with age, whereas the simple type of melanin decreases [24,25]. Consequently, melanin cannot be assumed as the only pigment in the RPE that causes depolarization.

One additional factor is that the RPE cell density and cell thickness is not constant and changes with retinal location [37]. The highest cell density and thickest cells can be found around the fovea and both parameters decrease towards the periphery, which represents a similar pattern as observed for depolarization. An increased thickness and cell density of the RPE is associated with increased melanin concentration [37] and as such can partly be the cause for the observed stronger depolarization. However, the true thickness of the RPE cannot be measured by our OCT system because of the limited axial resolution that lies in the range of the RPE cell thickness.

Throughout the present study, a kernel size of 10 × 10 pixels (corresponding to approximately 20 × 78 μm^2^ [axial × lateral]) was used to calculate DOPU [19]. This size has been chosen empirically by considering the trade-off between sensitivity to depolarization and resolution of the DOPU images. As reported in a previous study [38], the kernel size used to calculate DOPU needs to be large enough to contain a sufficiently large number of pixels that are above the noise level in the evaluation window and thus included into the calculation. There is a certain susceptibility to changes in this processing parameters and the kernel size used for DOPU calculation will influence the quantitative values of DOPU [14]. Nevertheless, this will only result in a shift to slightly lower or higher DOPU values but not in a change of the overall trends such as the DOPU dependency on age that have been reported here.

## Conclusion

In conclusion, we presented measurements of depolarization caused by the RPE in healthy and glaucoma subjects over a large field of view and investigated age and sex related changes. While there was no significant difference between the two study populations and between males and females, an increase of depolarization was found with age. In addition, depolarization depended on the retinal location and showed lower depolarization (higher DOPU values) at larger eccentricities from the fovea. Together with previous findings our results pave the way for further studies in other patient populations such as early-stage age-related macular degeneration, where potential changes in depolarization of the RPE introduced by the disease may be present.

## References

[1] Hu DN, Simon JD, Sarna T. Role of ocular melanin in ophthalmic physiology and pathology. Photochemistry and Photobiology. 2008;84(3):639–44. doi: 10.1111/j.1751-1097.2008.00316.x ISI:000255518400015. 18346089

[2] Anderson DH, Fisher SK, Steinberg RH. Mammalian Cones—Disk Shedding, Phagocytosis, and Renewal. Investigative Ophthalmology & Visual Science. 1978;17(2):117–33. ISI:A1978EQ25900004. 415019

[3] Kevany BM, Palczewski K. Phagocytosis of Retinal Rod and Cone Photoreceptors. Physiology. 2010;25(1):8–15. ISI:000274287300002. doi: 10.1152/physiol.00038.2009 20134024 PMC2839896

[4] Brown EE, DeWeerd AJ, Ildefonso CJ, Lewin AS, Ash JD. Mitochondrial oxidative stress in the retinal pigment epithelium (RPE) led to metabolic dysfunction in both the RPE and retinal photoreceptors. Redox Biol. 2019;24:101201. Artn 101201 ISI:000471255400032. doi: 10.1016/j.redox.2019.101201 31039480 PMC6488819

[5] Jonnal RS, Kocaoglu OP, Zawadzki RJ, Lee SH, Werner JS, Miller DT. The cellular origins of the outer retinal bands in optical coherence tomography images. Invest Ophthalmol Vis Sci. 2014;55(12):7904–18. Epub 2014/10/18. doi: 10.1167/iovs.14-14907 ; PubMed Central PMCID: PMC4261632. 25324288 PMC4261632

[6] Spaide RF, Curcio CA. Anatomical Correlates to the Bands Seen in the Outer Retina by Optical Coherence Tomography Literature Review and Model. Retina-the Journal of Retinal and Vitreous Diseases. 2011;31(8):1609–19. ISI:000294456100021. 10.1097/IAE.0b013e3182247535 PMC3619110 21844839

[7] Willoughby AS, Ying GS, Toth CA, Maguire MG, Burns RE, Grunwald JE, et al. Subretinal Hyperreflective Material in the Comparison of Age-Related Macular Degeneration Treatments Trials. Ophthalmology. 2015;122(9):1846–53. doi: 10.1016/j.ophtha.2015.05.042 ISI:000360116900025. 26143666 PMC4549177

[8] Pircher M, Hitzenberger CK, Schmidt-Erfurth U. Polarization sensitive optical coherence tomography in the human eye. Prog Retin Eye Res. 2011;30(6):431–51. Epub 2011/07/07. doi: 10.1016/j.preteyeres.2011.06.003 ; PubMed Central PMCID: PMC3205186. 21729763 PMC3205186

[9] de Boer JF, Hitzenberger CK, Yasuno Y. Polarization sensitive optical coherence tomography—a review [Invited]. Biomedical Optics Express. 2017;8(3):1838–73. doi: 10.1364/BOE.8.001838 ISI:000395942600042. 28663869 PMC5480584

[10] Pircher M, Götzinger E, Leitgeb R, Sattmann H, Findl O, Hitzenberger CK. Imaging of polarization properties of human retina in vivo with phase resolved transversal PS-OCT. Optics Express. 2004;12(24):5940–51. ISI:000225459700016. doi: 10.1364/opex.12.005940 19488235

[11] Pircher M, Götzinger E, Findl O, Michels S, Geitzenauer W, Leydolt C, et al. Human macula investigated in vivo with polarization-sensitive optical coherence tomography. Investigative Ophthalmology & Visual Science. 2006;47(12):5487–94. doi: 10.1167/iovs.05-1589 ISI:000242404900049. 17122140

[12] Miura M, Yamanari M, Iwasaki T, Elsner AE, Makita S, Yatagai T, et al. Imaging polarimetry in age-related macular degeneration. Investigative Ophthalmology & Visual Science. 2008;49(6):2661–7. doi: 10.1167/iovs.07-0501 ISI:000256306800049. 18515594 PMC3375125

[13] Ahlers C, Götzinger E, Pircher M, Golbaz I, Prager F, Schutze C, et al. Imaging of the Retinal Pigment Epithelium in Age-Related Macular Degeneration Using Polarization-Sensitive Optical Coherence Tomography. Investigative Ophthalmology & Visual Science. 2010;51(4):2149–57. doi: 10.1167/Iovs.09-3817 ISI:000275995800046. 19797228 PMC3016608

[14] Lippok N, Braaf B, Villiger M, Oh WY, Vakoc BJ, Bouma BE. Quantitative depolarization measurements for fiber-based polarization-sensitive optical frequency domain imaging of the retinal pigment epithelium. Journal of Biophotonics. 2019;12(1). ARTN e201800156 ISI:000455115500021. doi: 10.1002/jbio.201800156 30009506 PMC6526942

[15] Azuma S, Makita S, Kasaragod D, Sugiyama S, Miura M, Yasuno Y. Clinical multi-functional OCT for retinal imaging. Biomedical Optics Express. 2019;10(11):5724–43. doi: 10.1364/BOE.10.005724 ISI:000493997700020. 31799043 PMC6865108

[16] Kitano M, Fujita A, Asaoka R, Inoue T, Amari T, Komatsu K, et al. Assessment of macular function in patients with non-vascularized pigment epithelial detachment. Sci Rep-Uk. 2021;11(1). Artn 16577 ISI:000686663200038. doi: 10.1038/s41598-021-96151-8 34400749 PMC8368018

[17] Baumann B, Baumann SO, Konegger T, Pircher M, Gotzinger E, Schlanitz F, et al. Polarization sensitive optical coherence tomography of melanin provides intrinsic contrast based on depolarization. Biomedical Optics Express. 2012;3(7):1670–83. ISI:000306111400016. doi: 10.1364/BOE.3.001670 22808437 PMC3395490

[18] Yamanari M, Mase M, Obata R, Matsuzaki M, Minami T, Takagi S, et al. Melanin concentration and depolarization metrics measurement by polarization-sensitive optical coherence tomography. Sci Rep-Uk. 2020;10(1):19513. Artn 19513 ISI:000594818600020. doi: 10.1038/s41598-020-76397-4 33177585 PMC7658243

[19] Götzinger E, Pircher M, Geitzenauer W, Ahlers C, Baumann B, Michels S, et al. Retinal pigment epithelium segmentation by polarization sensitive optical coherence tomography. Optics Express. 2008;16(21):16410–22. doi: 10.1364/oe.16.016410 18852747 PMC2976032

[20] Lippok N, Villiger M, Bouma BE. Degree of polarization (uniformity) and depolarization index: unambiguous depolarization contrast for optical coherence tomography. Optics Letters. 2015;40(17):3954–7. doi: 10.1364/Ol.40.003954 ISI:000360810200009. 26368685 PMC4586115

[21] Fujita A, Amari T, Ueda K, Azuma K, Inoue T, Komatsu K, et al. Three-Dimensional Distribution Of Fundus Depolarization and Associating Factors Measured Using Polarization-Sensitive Optical Coherence Tomography. Transl Vis Sci Techn. 2021;10(2):Art 30:1–12. Artn30 ISI:000632914000003. doi: 10.1167/tvst.10.2.30 34003915 PMC7900852

[22] Baumann B, Götzinger E, Pircher M, Hitzenberger CK. Measurements of depolarization distribution in the healthy human macula by polarization sensitive OCT. Journal of Biophotonics. 2009;2(6–7):426–34. doi: 10.1002/jbio.200910031 ISI:000268690000018. 19526468

[23] Boulton M, Docchio F, Dayhawbarker P, Ramponi R, Cubeddu R. Age-Related-Changes in the Morphology, Absorption and Fluorescence of Melanosomes and Lipofuscin Granules of the Retinal-Pigment Epithelium. Vision Research. 1990;30(9):1291–303. doi: 10.1016/0042-6989(90)90003-4 ISI:A1990DU60200003. 2219746

[24] Feeneyburns L, Hilderbrand ES, Eldridge S. Aging Human Rpe—Morphometric Analysis of Macular, Equatorial, and Peripheral Cells. Investigative Ophthalmology & Visual Science. 1984;25(2):195–200. ISI:A1984SE86200010. 6698741

[25] Pollreisz A, Neschi M, Sloan KR, Schmidt-Erfurth U, Curcio CA, Dacey DM. Visualizing melanosomes (M) in apical processes (AP) of human retinal pigment epithelium (RPE) using volumetric serial block-face scanning electron microscopy (SBFSEM). Investigative Ophthalmology & Visual Science. 2018;59(9). ISI:000442912504132.

[26] Weinreb RN, Aung T, Medeiros FA. The Pathophysiology and Treatment of Glaucoma A Review. Jama-J Am Med Assoc. 2014;311(18):1901–11. doi: 10.1001/jama.2014.3192 ISI:000335798100022. 24825645 PMC4523637

[27] Sugita M, Zotter S, Pircher M, Makihira T, Saito K, Tomatsu N, et al. Motion artifact and speckle noise reduction in polarization sensitive optical coherence tomography by retinal tracking. Biomedical Optics Express. 2014;5(1):106–22. doi: 10.1364/Boe.5.5.000106 ISI:000329225500010. PMC3891324 24466480

[28] (IEC) IEC. Safety of Laser Products—Part 1: Equipment Classification and Requirements. Comission, editor2014.

[29] Schwarzhans F, Desissaire S, Steiner S, Pircher M, Hitzenberger CK, Resch H, et al. Generating large field of view en-face projection images from intra-acquisition motion compensated volumetric optical coherence tomography data. Biomedical Optics Express. 2020;11(12):6881–904. doi: 10.1364/BOE.404738 ISI:000596313900008. 33408968 PMC7747913

[30] Götzinger E, Baumann B, Pircher M, Hitzenberger CK. Polarization maintaining fiber based ultra-high resolution spectral domain polarization sensitive optical coherence tomography. Optics Express. 2009;17(25):22704–17. ISI:000272761300047. doi: 10.1364/OE.17.022704 20052196 PMC2963062

[31] Li K, Wu XD, Chen DZ, Sonka M. Optimal surface segmentation in volumetric images—A graph-theoretic approach. Ieee T Pattern Anal. 2006;28(1):119–34. doi: 10.1109/TPAMI.2006.19 ISI:000233172000010. 16402624 PMC2646122

[32] Garvin MK, Abramoff MD, Wu XD, Russell SR, Burns TL, Sonka M. Automated 3-D Intraretinal Layer Segmentation of Macular Spectral-Domain Optical Coherence Tomography Images. Ieee Transactions on Medical Imaging. 2009;28(9):1436–47. doi: 10.1109/TMI.2009.2016958 ISI:000269525300009. 19278927 PMC2911837

[33] Cunha-Vaz J, Ribeiro L, Lobo C. Phenotypes and biomarkers of diabetic retinopathy. Progress in Retinal and Eye Research. 2014;41:90–111. doi: 10.1016/j.preteyeres.2014.03.003 ISI:000338604100005. 24680929

[34] Harimoto A, Obata R, Yamamoto M, Aoki N, Yamanari M, Sugiyama S, et al. Retinal pigment epithelium melanin distribution estimated by polarisation entropy and its association with retinal sensitivity in patients with high myopia. British Journal of Ophthalmology. 2021. doi: 10.1136/bjophthalmol-2021-318890 ISI:000727747800001. 33958321

[35] Chen Q, Huang SH, Ma QK, Lin HL, Pan MM, Liu XT, et al. Ultra-high resolution profiles of macular intra-retinal layer thicknesses and associations with visual field defects in primary open angle glaucoma. Sci Rep-Uk. 2017;7. Artn 41100 ISI:000393504600001. doi: 10.1038/srep41100 28169283 PMC5294583

[36] Pollreisz A, Neschi M, Sloan KR, Pircher M, Mittermueller T, Dacey DM, et al. Atlas of Human Retinal Pigment Epithelium Organelles Significant for Clinical Imaging. Investigative Ophthalmology & Visual Science. 2020;61(8):Art13. Artn 13 ISI:000554499000004. doi: 10.1167/iovs.61.8.13 32648890 PMC7425708

[37] Boulton M, Dayhaw-Barker P. The role of the retinal pigment epithelium: topographical variation and ageing changes. Eye. 2001;15:384–9. doi: 10.1038/eye.2001.141 ISI:000169935800052. 11450762

[38] Sugita M, Pircher M, Zotter S, Baumann B, Saito K, Makihira T, et al. Analysis of optimum conditions of depolarization imaging by polarization-sensitive optical coherence tomography in the human retina. Journal of Biomedical Optics. 2015;20(1):016011. Artn 016011. doi: 10.1117/1.JBO.20.1.016011 ISI:000350206400020. 25585024

